# Quality of individual and group level interventions for first-episode psychosis at the tertiary psychiatric hospital in Uganda

**DOI:** 10.4102/sajpsychiatry.v27i0.1604

**Published:** 2021-04-22

**Authors:** Emmanuel K. Mwesiga, Noeline Nakasujja, Lawrence Nankaba, Juliet Nakku, Seggane Musisi

**Affiliations:** 1 Department of Psychiatry, College of Health Sciences, Makerere University, Kampala, Uganda; 2 Butabika National Referral Mental Hospital, Kampala, Uganda

**Keywords:** early intervention services, low and middle income country, public health, service provision, first episode psychosis, individual level interventions, group level interventions

## Abstract

**Background:**

Individual and group level interventions have the largest effect on outcomes in patients with the first episode of psychosis. The quality of these individual and group level interventions provided to first-episode psychosis patients in Uganda is unclear.

**Aim:**

To determine the quality of the individual and group level interventions provided to first episode psychosis patients in Uganda

**Setting:**

The study was performed at the only tertiary psychiatric hospital in Uganda.

**Methods:**

A retrospective chart review of recently discharged adult in-patients with the first episode of psychosis was performed. The proportion of participants who received different essential components for individual and group level interventions were calculated. From the different proportions, the quality of the services across the individual and group interventions was determined using the first-episode psychosis services fidelity scale (FEPS-FS). The FEPS-FS assigns a grade of 1–5 on a Likert scale depending on the proportion of patients who received the different components of the intervention. Twelve essential components across the individual and group interventions were assessed and their quality quantified.

**Results:**

The final sample included 156 first-episode psychosis patients. The median age was 27 years (inter-quartile range [IQR] [24–36]) and 55% of the participants were female. All 12 essential components had poor quality with the range of scores on the FEPS-FS between one and three. Only one essential component assessed (use of single antipsychotics) had moderate quality.

**Conclusion:**

Amongst current services at the Butabika National Referral Mental Hospital in Uganda, the essential components for individual and group level interventions for psychotic disorders are of poor quality. Further studies are required on how the quality of these interventions can be improved.

## Introduction

Psychotic disorders that include schizophrenia spectrum and bipolar disorders, if left untreated are associated with poor physical, psychological, social and occupational outcomes.^1,2,3,4^ Psychotic disorders follow an often common course of a premorbid phase, the prodrome, first-episode psychosis, a chronic relapsing phase and finally social impairment.^5^ Current treatment guidelines recommend for the early management of psychotic disorders at the first episode of psychosis.^6,7^ Early management, at the first episode of psychosis, has been associated with greater cognitive functioning, improved social functioning, fewer relapses and improved quality of life.^6,8^ Often early management for psychotic disorders is provided through specialised early intervention services (SEIS) which provide various interventions to ensure improved outcomes.^9,10^

In their seminal paper, Addington et al. described six key interventions that must be provided to patients with a first episode of psychosis to ensure improved patient outcomes.^11^ These interventions often provided in SEIS for psychotic disorders included; (1) population-level intervention and access; (2) comprehensive assessments and care plan; (3) individual-level interventions; (4) group-level interventions; (5) service system and models of intervention, and (6) evaluation and quality improvement.^11,12^ Within each intervention were essential components that varied in their effectiveness in improving patient outcomes.^11^ Addington et al. thus defined a grading system for the effectiveness of different essential components on patient outcomes.^11^ Grade ‘A’ implied there was strong evidence to support the component on improving patient outcomes. Grade ‘B’ implied there was supportive evidence of the component on improving patient outcomes. Grade ‘C’ implied this was just an opinion of clinicians on the effect of the component improving patient outcomes. Grade ‘D’ implied there was no evidence of benefit or harm of a component improving patient outcomes.^11,13^ An adaptation of their original 31 components, as well as the effectiveness of each component in improving outcomes, are highlighted in Table 1 below.

**TABLE 1:** Description and strength of essential components for early intervention services.

Intervention and essential components	Effectiveness of component in improving patient outcomes
**Population-level intervention and access**	
Targeted public education	B
Targeted education for health and social service providers	B
Acceptance of referrals with potential comorbid substance use disorders	C
Communication protocol between inpatient units and first-episode psychosis services	D
Timely contact with referred individual	D
**Comprehensive assessments and care plan**	
Individual-centred assessments	C
Comprehensive assessment upon enrolment	C
Assessment of suicidal thinking and behaviour	B
Care plan addresses psychosocial needs	C
Informed decision-making	C
Informed consent	D
**Individual-level interventions**	
**Pharmacotherapy**	
Selection of antipsychotic medication	A
Mode of antipsychotic administration	C
Low-dose, slow-increment antipsychotic medication	A
Clozapine for treatment resistance	A
Use of single antipsychotics	A
Monitoring metabolic changes	B
Monitoring antipsychotic medication side effects	C
Proactive steps to prevent weight gain and metabolic effects	B
**Psychoeducation, Individual**	
Individual psychoeducation	B
**Addictions treatment**	
Integrated mental health and addictions treatment	C
**Vocational and educational plans**	
Vocational plan	C
Supported employment	A
**Group-level interventions**	
Multifamily group psychoeducation	A
Group family psychoeducation	B
**Service systems and models of intervention**	
Psychiatrist as part of the team	C
Duration of first-episode psychosis services	B
Supervision and education of first-episode psychosis services staff	C
Weekly team meetings	B
Active outreach services	C
Crisis intervention services	C
**Evaluation and quality improvement**	
Tracking of process and outcome measures	C

*Source*: Adapted from Addington DE, McKenzie E, Norman R, Wang J, Bond GR. Essential evidence-based components of first-episode psychosis services. Psychiatr Serv. 2013;64(5):452–457. https://doi.org/10.1176/appi.ps.201200156

A, strong evidence; B, supportive evidence; C, opinion; D, no evidence of benefit or harm.

Individual and group level interventions are the most effective interventions for patients with first-episode psychosis.^12,14^ They are the only interventions with essential components that have an effectiveness grade of ‘A’ (there is strong evidence of the essential components’ effect on patient outcomes).^11^

Individual-level interventions have 12 components and 5 of them (selection of antipsychotic medication, supported employment, use of single antipsychotics, low dose slow increment for antipsychotic medication, clozapine for treatment resistance) have an effectiveness grade of ‘A’.^6,15,16^ Group-level interventions have only two components: one (multifamily group psychoeducation) is graded ‘A’ for effectiveness on outcomes, whilst the other (group family psychoeducation) is rated ‘B’ (supportive evidence) for effectiveness on outcomes for patients with psychotic disorders.^17,18^

We aimed to determine the quality of individual and group level interventions provided to patients with a first episode of psychosis at Butabika National Referral Mental Hospital in Uganda, also known as Butabika Hospital. Even though there are no SEIS for psychotic disorders at the hospital, we hypothesised that individual and group level interventions of SEIS for psychotic disorders are provided within routine care. It is however unclear if the individual and group level interventions provided are of the prerequisite quality. Given that the largest mental illness burden in this setting is for psychotic disorders,^19^ the interventions that have the biggest impact on patient outcomes must be provided with the utmost quality.

## Method

### Study design and setting

This was a retrospective chart review performed at Butabika National Referral Mental Hospital in Uganda. It is the only national psychiatric hospital in Uganda. It is also responsible for directing mental health policy and financing, issuing directives on which interventions are to become standard care in the country.

There are specialised clinics for addiction, child and adolescent mental health, occupational therapy and human immunodeficiency virus/acquired immunodeficiency syndrome (HIV/AIDS) mental health. Although psychotic disorders are the most common disorders at Butabika Hospital,^19^ there are currently no SEIS for psychotic disorders.

### Study participants

Participants were recruited from a larger study on predictors of cognitive impairment amongst patients with the first episode of psychosis. The participants were all antipsychotic naïve at enrollment, aged 18–60 years, without substance use history, HIV/AIDS and/or syphilis. This retrospective chart review included all participants enrolled in the initial study.

### Tools

#### The first-episode psychosis services fidelity scale

The quality of the services provided for each component of the specialised service was assessed using the First-Episode Psychosis Services Fidelity Scale (FEPS-FS).^20^ This is an evidence-based practice and rating criteria used to assess the degree to which programmes deliver evidence-based practices.^21^ The tool assesses the extent to which a service in its entirety is providing evidence-based interventions. It does not assess how many participants are receiving the service but rather the overall standard of care being provided.

A Likert scale from 1 to 5 determines how well the standard of care is with a score of 1 if the target component is met in 0% – 19% of patients, 2 in 20% – 39% of patients, 3 in 40% – 59% of patients, 4 in 60% – 79% of patients and 5 if the target component is met in greater than 80% of patients. Ratings of four and above imply satisfactory performance.^21^

### Study procedure

A master list of all the patients previously enrolled was made, and the files of the participants were obtained from the hospital records office. Files were reviewed from the period of the participant’s first admission. The different essential components of individual and group level interventions received by each participant were recorded till discharge. This data was captured in a spreadsheet specifically designed to assess the interventions and essential components that were available. For each essential component assessed, a ‘yes’/’no’ was recorded in the excel sheet depending on whether the participant had or had not received the essential component. If the research assistant could not tell if the essential component had been provided to the participant, the research assistant reported ‘unclear’. A summary excel sheet can be found in the supplementary files. The specific criteria to determine if an essential component had been received are highlighted is provided in Table 2. The questions used to determine if a component was available were developed from the literature on essential components by Addington.^11^ Research assistants were psychiatric clinical officers trained before the onset of the study on how to assess for the different criteria in Addington’s checklist from the patient file.

**TABLE 2:** Summary of how the essential, evidence-based components for specialised early intervention services were assessed in the study.

Component	Description in the study
**Individual-level interventions**	
**Pharmacotherapy**	
Selection of antipsychotic medication	FGA versus SGA. Second-generation antipsychotics preferred.
Mode of antipsychotic administration	Oral, parental or both. Oral preferred initially with the depot in the long term.
Low-dose, slow-increment antipsychotic medication	Assessed whether a low dose of any antipsychotic was started with subsequent increments were necessary.
Clozapine for treatment resistance	Not applicable as this was a first episode population.
Use of single antipsychotics	Used one or multiple antipsychotics.
Monitoring metabolic changes	BMI, cholesterol, RBS, weight gain.
Monitoring antipsychotic medication side effects	Notes reporting side effects.
Proactive steps to prevent weight gain and metabolic effects	Recommended exercise in notes.
**Psychoeducation, individual**	
Individual psychoeducation	Clinical psychologist review.
**Addictions treatment**	
Integrated mental health and addictions treatment	Not assessed, as substance use disorder was an exclusion criterion in the previous study.
**Vocational and educational plans**	
Vocational plan	Social worker review.
Supported employment	Plan for supported employment.
**Group-level interventions**	
Multifamily group psychoeducation	Meetings with families who were taught about the illness.
Group family psychoeducation	Family sessions.

Source: Developed from the literature on essential components by Addington DE, McKenzie E, Norman R, Wang J, Bond GR. Essential evidence-based components of first-episode psychosis services. Psychiatr Serv. 2013;64(5):452–457. https://doi.org/10.1176/appi.ps.201200156

FGA, first-generation antipsychotics; SGA, second-generation antipsychotics; BMI, body mass index; RBS, random blood sugar.

### Statistical analyses

The information abstracted from the chart review was merged by study identification numbers to the original dataset on predictors of cognitive impairment amongst patients with the first episode of psychosis. Data was analysed using Stata version 14.0.^22^ Descriptive statistics were employed to determine the proportion of patients receiving each essential component. The proportions of patients receiving a specific essential component were then used to determine the quality of the essential component provided.

### Ethical considerations

As this was a retrospective chart review of file records, we did not receive individual patient consent. In the primary study, participants were asked if they could be contacted for enrolment in future studies. This chart review received institutional approval from the hospital administration. The original study from which the patients were recruited received ethical approval from the Uganda National Council for Science and Technology (UNCST), the School of Medicine Research and Ethics Committee (SOMREC) of Makerere University, ethical clearance reference number: 2017-086, and institutional approval from Butabika National Referral Mental Hospital.

## Results

The charts of 156 participants were reviewed. Participants had been enrolled in the previous study between January 2018 and January 2019. The median age of the participants was 27 years (IQR 24–36). Most (84/156 [55.3%]) of the participants were female. The majority (83.6%) of participants were either unemployed or in non-formal employment. Most (69%) of participants reported that this was their first time of experiencing symptoms and 40% had consulted an alternative and complementary therapist (traditional healer, religious healer) before admission at the hospital. Other characteristics of the patients are highlighted in Table 3 below.

**TABLE 3:** Baseline characteristics of the participants.

Factor	Level	Frequency		Median	IQR
		*n*	%		
Age	Median	-	-	27	24–36
Gender	Male	68	44.7	-	-
	Female	84	55.3	-	
Marital status	Single	76	50.0	-	-
	Married	47	30.9	-	-
	Divorced	29	19.1	-	-
Current employment history	Student	12	7.9	-	-
	Formal employment	13	8.6	-	-
	Non-formal employment	63	41.5	-	-
	Unemployed	64	42.1	-	-
Highest level of education	No school	4	2.6	-	-
	Primary	62	40.8	-	-
	Secondary	65	42.8	-	-
	Diploma	17	11.2	-	-
	University	4	2.6	-	-
Ethnic	Bantu	120	80.0	-	-
	Nilotic	10	6.7	-	-
	NiloHamites	5	3.3	-	-
	Sudanic	5	3.3	-	-
	Hamites	10	6.7	-	-
Diagnosis	Affective psychosis	53	49.1	-	-
	Non-affective psychosis	55	50.9	-	-
Age first seeking help for psychosis	Median	-	-	25	21–29
First presentation at the medical facility	No	12.7	12.7	-	-
	Yes	87.3	87.3	-	-
Previous use of alternative therapy	No	35.1	35.1	-	-
	Yes	64.9	64.9	-	-

IQR, inter-quartile range.

Most service components were of poor quality as the proportion of patients receiving the component was few. The proportions of patients who received different individual and group level interventions as well as the quality of the services for each component are highlighted in Table 4 below.

**TABLE 4:** Quality of essential components for individual and group level interventions.

Essential component	Effectiveness of component in improving patient outcomes	Proportion of participants who had received the component		Quality of service as assessed by FEPS-FS Range (1–5)
		*N*	%	
**Individual-level interventions Pharmacotherapy**				
Selection of antipsychotic medication	A	Both 27	16.98	01
		FGA 129	81.13	
		SGA 03	1.89	
Mode of antipsychotic administration	C	Oral 52	32.70	02
		Parenteral 01	0.63	
		Both 106	66.67	
Low-dose, slow-increment antipsychotic medication	A	Yes 05	3.14	01
		No 152	95.60	
		Unclear 02	1.26	
Clozapine for treatment resistance	A	N/A	-	N/A
Use of single antipsychotics	A	Yes 78	49.68	03
		No 79	50.32	
Monitoring metabolic changes	B	Yes 04	2.5	01
		No 156	97.5	
Monitoring antipsychotic medication side effects	C	Yes 39	24.68	02
		No 119	75.32	
Proactive steps to prevent weight gain and metabolic effects	B	Yes 02	1.26	01
		(Diet = 01)		
		(Exercise = 01)		
		No 157	98.74	
**Psychoeducation, Individual**				
Individual psychoeducation	B	Yes 36	22.78	02
		No 122	77.22	
**Addictions treatment**				
Integrated mental health and addictions treatment	C	N/A	-	N/A
**Vocational and educational plans**				
Vocational plan	C	Yes 04	2.53	01
		No 154	97.47	
Supported employment	A	Yes 01	0.63	01
		No 157	99.37	
**Group-level interventions**				
Multifamily group psychoeducation	A	Yes 22	13.92	01
		No 136	86.08	
Group family psychoeducation	B	Yes 21	13.29	01
		No 137	86.71	

FGA, first-generation antipsychotics; SGA, second-generation antipsychotics; FEPS-FS, first-episode psychosis services fidelity scale; N/A, not applicable; A, strong evidence; B, supportive evidence; C, opinion.

## Discussion

In confirming our hypothesis, we found that Butabika National Referral Mental Hospital in Uganda already provides individual and group level interventions for patients with the first episode of psychosis. The quality of essential components is however poor.

Of the six interventions that had been given an ‘A’ rating in the Addington checklist (selection of antipsychotic medication, supported employment, use of single antipsychotics, low dose slow increment for antipsychotic medication, clozapine for treatment resistance and multifamily group psychoeducation), only one (use of single antipsychotics) reached the level of moderate quality service provision in this setting. The proportion of patients who used only one antipsychotic was similar to literature from high-income countries (HIC).^23,24^ Whether this was by design or because of a limited selection of antipsychotic drugs is not clear from the study. Mental health services in low resource settings are often plagued by minimal financing which might point to limited drug selection.^25,26^ In Uganda, for example, there is limited availability of second-generation antipsychotics (SGA).^27^

Three other interventions with ‘A’ rating (selection of antipsychotic medication, supported employment, low dose slow increment for antipsychotic medication) were of poor quality. The poor quality in the component of slow dose increment of antipsychotic medication could be because of various factors. First, we are cognisant of the fact that initial doses for initiation of treatment are dependent on the severity of the illness.^28^ In our setting, Abbo et al. highlighted that patients with psychotic disorders often present late with severe illness after attempting alternative treatments.^29,30,31,32^ There might therefore be a need to initiate treatment at higher doses than is recommended. Second, it might also not be possible to make slow increments because of the lack of formulations of antipsychotic medications available in low resource settings.^27^ Finally, given the brief time frame of the study (admission to discharge), it is possible that the increments were made over a longer period. This study, therefore, needs replication by longitudinal studies to clearly define the dose adjustments over 2 years.

Selection of antipsychotic medication had most participants receiving first-generation antipsychotics (FGAs) which are cheaper even though SGA are preferred.^27^ This is like literature from low resource settings whilst HIC often use SGA. These FGAs are associated with a greater side effect profile leading to poor drug adherence. Multifamily group psychoeducation and supported employment have also been shown to have strong evidence of good outcomes for patients with psychotic disorders. These services are unfortunately still of poor quality at Butabika hospital as there are a limited number of therapists employed by the hospital.^19^ This may be because of human resource and financing limitations which are common in low resource settings like Uganda.^26,33,34,35^ The lack of patients being supported in employment is because of the general lack of social support services in Uganda. These supported employment programmes also require extensive resources and personnel that are not available in low resource settings like Uganda.^36,37^

Four interventions were rated as having supportive evidence (rating B) for improved outcomes by Addington et al.^11^ These included monitoring of metabolic side effects, individual psychoeducation, group psychoeducation and proactive steps to promote exercise and prevent weight gain. That there was moderate quality of the individual psychoeducation intervention is noteworthy. This is because SEIS for psychotic disorders not only target first-episode psychosis patients but also those in the psychosis prodrome, as well as high-risk and ultra-high risk individuals.^6^ This psychoeducation is therefore a tool that can be used in the prevention of the psychosis onset, or early initiation of treatment. The available services were of poor quality for monitoring side effects and preventing weight gain. There is a need for improved awareness of the increased risk for non-communicable diseases in patients with psychotic disorders, even in patients on first-generation antipsychotic medication.^38^ Increased promotion of exercise will be required as the hospital adapts SGA as the mainstay of treatment.^27^

The level of evidence for a vocational plan, mode of administration of antipsychotics and monitoring side effects was ‘C’ (opinion). All these interventions had poor to moderate quality of services in the hospital. In low resource settings like Uganda FGA are more available than SGA, FGA.^27^ These FGAs are often associated with worse side effects leading to poorer adherence and worse outcomes.^39,40^ We postulate that in low resource settings monitoring antipsychotic side effects may have a larger impact on the outcome than in HIC.^39^

### Study limitations

There have been many previous attempts to identify services essential for first-episode psychosis clinics.^11,16^ However, the Delphi processes and systematic reviews that identified these core components had limited representation from low- and middle-income countries. As such many of these processes may not be culturally specific, hence calling for pre-implementation cultural validation of the exercise. For example, given that many of the participants were unemployed or in non-formal employment, it is possible that vocational plans have a bigger impact on outcomes and therefore may need to be assigned higher levels of evidence in our setting. The same argument made for integrated addictions treatment has given the high rates of substance abuse in our setting, not just amongst patients with psychotic disorders but the general population as well.^41,42^ Further research is therefore needed to validate the low levels of evidence (opinion) for these interventions in low resource settings. This study however may form a basis for the development of SEIS for psychotic disorders in the region.

## Conclusions and recommendations

Our findings, in this study, showed that even within the everyday care in our low resource settings, there are essential components of SEIS for psychotic disorders that are available that could be implemented at Butabika National Referral Mental Hospital. There is a need however, for improvement in the quality of these services, especially in those components that showed the strongest evidence for improved outcomes in previous studies. Finally, long-term studies are needed to validate the level of evidence for these components given the human resource, financing and health-seeking behaviour differences between high-income and low-income countries, and in different cultural settings. Further research is also needed on the availability of other components like population-level interventions which are necessary for developing SEIS for psychotic disorders.^43^
